# Associations of blood biomarkers with arterial stiffness in patients with diabetes mellitus: A population‐based study

**DOI:** 10.1111/1753-0407.13433

**Published:** 2023-06-16

**Authors:** Yong‐Qi Liang, Rui Zhou, Hao‐Wen Chen, Bi‐Fei Cao, Wei‐Dong Fan, Kuan Liu, Qi Zhong, Yi‐Ning Huang, Xian‐Bo Wu, Meng‐Chen Zou

**Affiliations:** ^1^ Department of Epidemiology, School of Public Health Southern Medical University Guangzhou China; ^2^ Department of Endocrinology and Metabolism, Nanfang Hospital Southern Medical University Guangzhou China

**Keywords:** arterial stiffness, blood biomarkers, diabetes, mortality, 动脉硬化, 血液生物标志物, 糖尿病, 死亡率。

## Abstract

**Background:**

Arterial stiffness contributes to additional cardiovascular risks in diabetic patients by triggering the loss of vascular and myocardial compliance and promoting endothelial dysfunction. Thus, prevention of arterial stiffness is a public health priority, and the identification of potential biomarkers may provide benefits for early prevention. This study investigates the relationships between serum laboratory tests and pulse wave velocity (PWV) tests. We also investigated the associations between PWV and all‐cause mortality.

**Methods:**

We examined a panel of 33 blood biomarkers among diabetic populations in the Atherosclerosis Risk in Communities Study. The carotid‐femoral (cfPWV) and femoral‐ankle PWV (faPWV) were measured using an automated cardiovascular screening device. The aortic‐femoral arterial stiffness gradient (afSG) was calculated as faPWV divided by cfPWV. Biomarker levels were log‐transformed and correlated with PWV. Cox proportional hazard models were employed for survival analysis.

**Results:**

Among 1079 diabetic patients, biomarkers including high‐density lipoprotein cholesterol, glycated hemoglobin, high‐sensitivity troponin T, cystatin C, creatinine, and albuminuria were significantly correlated with afSG (*R* = 0.078, −0.193, −0.155, −0.153, −0.116, and −0.137, respectively) and cfPWV (*R* = −0.068, 0.175, 0.128, 0.066, 0.202, and 0.062, respectively). Compared with the lowest tertile of afSG, the risk of all‐cause mortality was lower in the highest tertile (hazard ratio 0.543; 95% confidence interval 0.328–0.900).

**Conclusion:**

Certain biomarkers related to blood glucose monitoring, myocardial injury, and renal function significantly correlated with PWV, suggesting that these putative risk factors are likely to be important atherosclerosis mechanisms in diabetic patients. AfSG may be an independent predictor of mortality among diabetic populations.

## Graphical abstract

Highlights

- Few studies have investigated the association of large‐scale biomarkers and pulse wave velocity (PWV) among diabetic patients.
- This study investigates the relationships of serum laboratory tests with PWV and explored the associations between PWV and all‐cause mortality among diabetic patients.
- Certain biomarkers related to blood glucose monitoring, myocardial injury, and renal function significantly correlated with PWV among diabetic patients.
- Our research was the first to demonstrate that higher aortic‐femoral arterial stiffness gradient was associated with lower all‐cause mortality in diabetic patients, independent of a wide range of covariates.

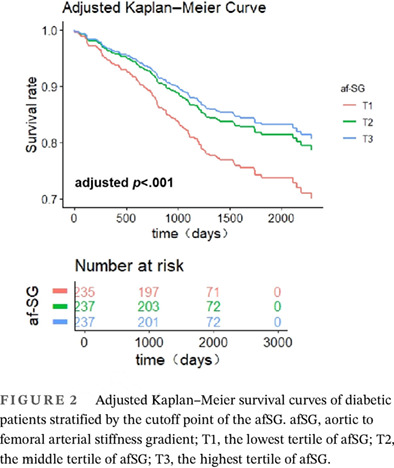

## 摘要

背景: 动脉僵硬导致糖尿病患者心血管疾病风险增加，其通过引发血管和心肌顺应性的下降以及促进内皮功能失调起作用。因此，预防动脉硬化成为公共卫生的优先事项，识别潜在的生物标志物可能对早期预防有益。本研究旨在探讨血清实验室检测与脉搏波速度(PWV)检测之间的关系，并研究PWV与全因死亡率之间的关系。

方法: 在动脉粥样硬化风险社区研究的糖尿病患者中，我们检测了一系列33种血液生物标志物。颈动脉‐股动脉PWV(cfPWV)和股动脉‐踝动脉PWV(faPWV)的PWV采用自动心血管筛查设备进行测量，主动脉‐股动脉的动脉硬化梯度(afSG)按faPWV除以cfPWV进行计算。将生物标志物水平进行对数转换后与PWV进行相关性分析。采用Cox比例风险模型进行生存分析。

结果: 在1,079名糖尿病患者中，包括高密度脂蛋白胆固醇、糖化血红蛋白、高敏肌钙蛋白T、胱抑素C、肌酐和尿白蛋白等生物标志物与afSG（R=0.078，‐0.193，‐0.155，‐0.153，‐0.116和‐0.137，分别为）和cfPWV（R=‐0.068，0.175，0.128，0.066，0.202和0.062）显著相关。与afSG的最低三分位数相比，最高三分位数的全因死亡风险较低（危险比0.543; 95%置信区间0.328‐0.900）。

结论: 与血糖监测、心肌损伤和肾功能相关的某些生物标志物与PWV显著相关，提示这些潜在的危险因素可能是糖尿病患者的关键动脉粥样硬化机制。AfSG可能是糖尿病患者死亡的独立预测指标。

## 1 INTRODUCTION

Atherosclerosis, a critical pathophysiological process in the progression of ischemic strokes, coronary heart disease, and other cardiovascular events, entails a potential disease burden that places enormous pressure on medical and socioeconomic systems.^1^ Long‐term persistent hyperglycemic status leads to various complications, and atherosclerotic cardiovascular disease is the main macrovascular complication of diabetes, with direct clinical consequences including an increased risk of stroke, left ventricular hypertrophy, and heart failure.^2^ Moreover, atherosclerotic cardiovascular disease is the leading cause of death and disability in patients with diabetes.^3^, ^4^ One of the main mechanisms of atherosclerosis is the formation of advanced glycation end‐products (AGEs), which may lead to loss of collagen elasticity and subsequent increase in arterial stiffness.^5^ In addition, excessive mitochondrial reactive oxygen species (ROS) production in the endothelium leads to endothelial dysfunction which may also contribute to the development of arterial stiffness related to diabetes.^6^, ^7^ Therefore, providing early interventions in atherosclerosis is vital for diabetic populations.

Pulse wave velocity (PWV), the most widely used measurement of arterial stiffness, is a known risk factor for the incidence of cardiovascular disease (CVD). In particular, the carotid‐femoral pulse wave velocity (cfPWV) test is the recommended gold standard for measuring arterial stiffness in clinical practice.^8^, ^9^ However, an inherent limitation of arterial stiffness measures, including cfPWV, is their high dependence on operational mean arterial pressure.^10^ Lower extremities represent a significant portion of the entire arterial system and are more prone to arteriosclerotic processes than the upper extremities.^11^ Thus, the aortic‐femoral arterial stiffness gradient (afSG), which additionally considers the lower extremities and provides a more comprehensive picture of hemodynamic integration, is considered as a more promising arterial stiffness measurement for evaluating the status of atherosclerosis.^11^, ^12^

Traditional diabetic risk factors, including hypertension,^13^ insulin resistance,^14^ and (central) obesity,^15^ are all considered contributors to arteriosclerosis, likely confounding much of the association between PWV and diabetes. However, unconventional risk factors directly result from arteriosclerosis, such as chronic inflammation,^16^ lipid metabolism,^17^ glycemic control,^18^ renal disease,^19^, ^20^ and thyroid function,^21^ are also proposed to contribute to the excess diabetic risk observed in populations with arteriosclerosis by inducing islet beta‐cell inflammation, impairing beta‐cell functions, and enhancing insulin resistance.^22^ Considerable evidence suggests that arterial stiffness is a predictor of cardiovascular events and mortality independent of traditional risk factors.^23^, ^24^, ^25^ Furthermore, despite the long‐standing observation between atherosclerosis and diabetes, limited studies have examined the relationship of biomarkers with PWV. Notably, no previous study in type 2 diabetes has evaluated whether afSG was predictive of all‐cause mortality. To fill this knowledge gap, we examined the correlations of biomarkers related to myocardial injury, inflammation, lipid metabolism, complete blood count, and renal or thyroid index with PWV using a large prospective population‐based study of patients with diabetes. We also aimed to determine if any associations between PWV and all‐cause mortality occur in diabetic patients.

## 2 METHODS

### 2.1 Study population

The Atherosclerosis Risk in Communities (ARIC) Study is a population‐based, longitudinal study of 15 792 men and women aged 45–64 years enrolled between 1987 and 1989 from four US communities (Forsyth County, North Carolina; Jackson, Mississippi; Minneapolis, Minnesota; and Washington County, Maryland). Participants underwent follow‐up clinical examinations every 3 years denoted as Wave 2 (1990–1992), Wave 3 (1993–1995), and Wave 4 (1996–1998) visits. Fifteen years later, 5850 participants were invited back for the following three visits: Wave 5 (2011–2013), Wave 6 (2016–2017), and Wave 7 (2018–2019). Details of the ARIC study have been described previously.^26^ The current analysis utilized data collected during Waves 5 and 6, and our study focused on participants diagnosed with diabetes. Diabetes was defined as fasting glucose ≥126 mg/dL, nonfasting glucose ≥200 mg/dL, or self‐reported diagnosis of diabetes by a physician.^11^, ^27^, ^28^, ^29^ Of 5850 participants who attended Wave 5 between 2011 and 2013, we excluded participants with missing PWV measurements (*n* = 1147), nondiabetic populations (*n* = 2554), those with coronary heart diseases (*n* = 902), and with missing biomarkers (*n* = 116) or covariates (*n* = 52), resulting in a final sample of 1079 (Figure S1 in Data S1). We further excluded 370 participants who lacked follow‐up data in survival analysis. ARIC was approved by the institutional review boards at all participating institutions, and all participants provided written informed consent at enrollment.

### 2.2 Experimental measures

#### 2.2.1 Pulse wave velocity

After participants stayed in the supine position for 5–10 min, technicians measured their cfPWV and femoral‐ankle PWV (faPWV) following a standardized protocol and using the automated cardiovascular screening device VP‐1000 Plus (Omron, Kyoto, Japan).^30^ CfPWV is calculated as the carotid to femoral distance minus the suprasternal notch to carotid distance. FaPWV measures peripheral arterial stiffness and is estimated as the distance between femoral and ankle sampling sites determined using height‐based formulas, divided by the time delay between femoral and ankle waveforms.^12^ A minimum of two PWV measurements were taken for both arms, and the last two measurements were averaged. The average of the left and right faPWV measures was included for analysis. The afSG was calculated by dividing faPWV by cfPWV. This method emphasizes the increased arterial stiffness between the central and distal arteries in a cardiovascular system. Although no clinical threshold has been identified, an afSG greater than 1.0 (ie, faPWV > cfPWV) was considered physiologically normal and an afSG of 1.0 or less (cfPWV ≥ faPWV) was considered pathological to ensure a wide context.^11^, ^12^

#### 2.2.2 Biomarkers

Each blood biochemistry test in the Wave 5 was categorized as either “lipid metabolism” (ie, total cholesterol, total triglycerides, high‐density lipoprotein cholesterol [HDL‐C], and low‐density lipoprotein cholesterol), “blood glucose monitoring” (ie, fasting glucose, insulin, insulin resistance index, and glycated hemoglobin [HbA1c]), “complete blood counts” (ie, white blood cell count, red blood cell count, hemoglobin, hematocrit [HCT], platelet count [PLT], mean cell volume, mean corpuscular hemoglobin, mean corpuscular hemoglobin concentration, red cell distribution width [RDW], mean platelet volume, lymphocyte percentage, monocyte percentage, and granulocyte percentage), “myocardial injury” (ie, N‐terminal pro‐B‐type natriuretic peptide and high‐sensitivity troponin T [hs‐TnT]), “inflammation” (ie, high sensitive C‐reactive protein [CRP]), “renal” (ie, creatinine, uric acid, estimated glomerular filtration rate [eGFR], albuminuria, urine creatinine, and cystatin C [CysC]), or “thyroid index” (ie, thyroxine (free), triiodothyronine, thyroid stimulating hormone, and thyroid peroxidase antibody). The Atherosclerosis Laboratory (Baylor College of Medicine in Houston, TX) performed assays related to lipid metabolism, blood glucose monitoring, complete blood counts, myocardial injury, and inflammatory indicators. The Clinical Chemistry Laboratory (the University of Minnesota in Minneapolis, MN) examined the renal and thyroid indexes. The details of specific equipment and materials used to measure each biomarker were obtained from ARIC (https://sites.cscc.unc.edu/aric/sites/default/files/public/manuals/Manual%208%20Laboratory%20Determinations%20revised%2009242012.pdf). Rigorous quality control and correction were performed for technical outliers by referencing the National Committee for Clinical Laboratory Standards (details available at https://sites.cscc.unc.edu/aric/sites/default/files/public/manuals/Manual%2012%20QA%20and%20QC.pdf).

### 2.3 Ascertainment of death

The secondary outcome was all‐cause mortality, subsequent to Wave 6 until the end of 2017. Deaths were determined with annual (or later, semiannual) telephone calls and linkage to local hospital and state health department records. The follow‐up period was calculated from the date of participation in Wave 5 until death or the end of the follow‐up, whichever occurred first.

### 2.4 Covariates

The following covariates were demonstrated in previous studies to be associated with carotid atherosclerosis risks among diabetic patients^31^ were selected for our analyses: age (continuous), sex (male and female), race (White and African Americans), smoking status (never, former, or current), drinking status (never, former, or current), body mass index (BMI, kg/m^2^), physical activity (yes or no), self‐reported disease history (stroke, myocardial infarction, heart failure, and cancer), and use of medications including antihypertensive drugs, hypoglycemic drugs, lipid‐lowering drugs, anticoagulants, and statins. Medication information was obtained from diagnosis by a physician or the 2004 Med Code.

### 2.5 Statistical analysis

To assess the normality of continuous data, we utilized the Kolmogorov–Smirnov test. Normally distributed data were reported as mean (SD). If normality was not observed, then we reported the data as median (interquartile range). Categorical data were reported as frequencies (*n*) and percentages (%). One‐way analysis of variance (ANOVA) and *χ* ^*2*^tests were used to test the significance of differences among more than two groups for continuous and categorical variables, respectively. The Kruskal–Wallis H test was used as a nonparametric alternative for ANOVA. Correlations of biomarker levels with afSG, cfPWV, and faPWV were calculated by Spearman rank correlations. To further evaluate potentially linear relationships, the biomarkers and PWV were then both log‐transformed and correlated using Pearson correlations.

To explore the association of PWV measurements with the risk of all‐cause mortality, multivariable‐adjusted Cox proportional hazards models (all model met the proportional‐hazards assumption, *p >* .05) were used to calculate the hazard ratios (HRs) and 95% confidence intervals (95% CIs) for all‐cause mortality according to tertiles of the cfPWV, faPWV, and afSG levels. Associations of PWV measurements with all‐cause mortality among diabetic patients were calculated for the unadjusted model, by adjusting for age and sex (Model 1), and adjusting for variables included in Model 1 plus race, smoking status, drinking status, BMI, physical activity, antihypertensive drugs, hypoglycemic drugs, lipid‐lowering drugs, anticoagulants, statins, stroke, myocardial infarction, heart failure, and cancer (Model 2). A sensitivity analyses was undertaken, and we analyzed the association between afSG and all‐cause mortality by excluding participants who developed CVDs or cancer in the follow‐up. We also performed subgroup analyses stratified by sex (male/female), age (<75 years or ≥75 years), race (white, African American), drinking status (current drinker or not), smoking status (current smoker or not), history of CVD (yes/no), use of antihypertensive drugs (yes/no), and hypoglycemic drugs (yes/no). The product items of the aforementioned layered factors and afSG were incorporated into multivariate Cox regression models (based on Model 2) as interaction terms to test whether an interaction occurs and evaluate the robustness of our main results.

All analyses were performed using Stata version 17.0, and figures were drawn by R version 4.2.2. All statistical tests described here were two sided, and differences at *p* < .05 were accepted as statistically significant.

## 3 RESULTS

### 3.1 Baseline characteristics

**FIGURE 1:**
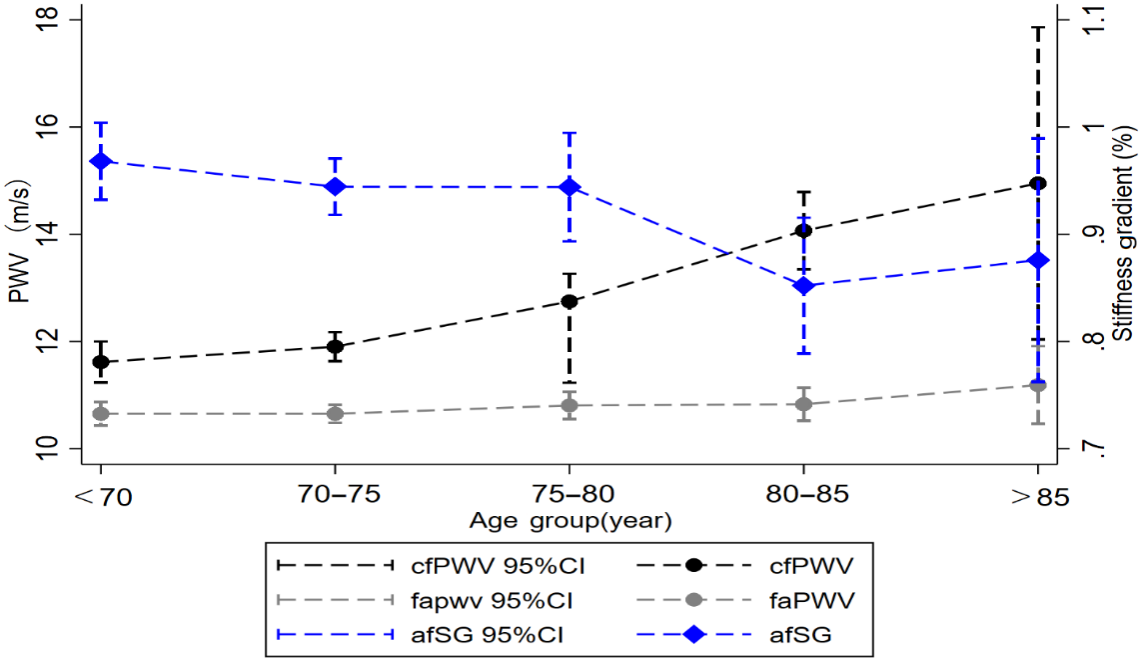
Mean carotid‐femoral pulse‐wave velocity (cfPWV), femoral‐ankle pulse‐wave velocity (faPWV), and aortic‐femoral arterial stiffness gradient (afSG) in 5‐year age groups, with 95% confidence intervals. Black dot and dashed line mean cfPWV and 95% CI; gray dot and dashed line mean faPWV and 95% CI; blue diamond and dashed line mean afSG and 95% CI. 95% CI, 95% confidence interval.

A total of 1079 eligible diabetic patients (mean age was 75.2 ± 5.0 years, 59.3% were females) with PWV measurements were recruited in Wave 5. Table S1 in Data S1 shows the baseline characteristics for all patients. The cfPWV, faPWV, and afSG values stratified by age categories are presented in Figure 1. Mean faPWV remained virtually unchanged at 10.80 m/s, but mean cfPWV increased and mean afSG decreased across age groups.

### 3.2 Correlations of biomarker levels with cfPWV, faPWV, and afSG

The median (interquartile range) levels of biomarkers according to afSG tertiles are described in Table 1. The levels of HbA1c, creatinine, uric acid, uric albumin, CysC, hs‐TnT, and CRP all decreased with increasing afSG (all *p* < 0.05), whereas HDL‐C levels increased (*p* = 0.04). The correlations between biomarker levels and PWV measurements were assessed using Spearman rank correlation analysis (Table 2). Moderate correlations were detected between HbA1c (*R* = 0.175), albuminuria (*R* = 0.202), hs‐TnT (*R* = 0.128), and cfPWV, and between fasting glucose (*R* = 0.102), creatinine (*R* = −0.116), CysC (*R* = −0.193), platelet count (*R* = 0.164), HCT (*R* = 0.149), RDW (*R* = −0.163), CRP (*R* = −0.133), and faPWV. Slight correlations were found for HbA1c, creatinine, CysC, albuminuria, RDW, hs‐TnT, and afSG (*R* = −0.193, −0.116, −0.153, −0.153, −0.116, −0.155, −0.108, respectively). Biomarker levels and PWV measurements (cfPWV, faPWV, and afSG) were both log‐transformed and subjected to Pearson correlation analysis, for which similar results were obtained.

**TABLE 1:** Median (IQR) levels of biomarkers according to the afSG category.

Biomarker	Overall	afSG tertiles			*p* value^a^
		T1	T2	T3	
Lipid metabolism–					
Total cholesterol, mg/dl	170.00 (146.00–199.00)	171.00 (145.00–204.00)	170.00 (147.00–198.00)	171.00 (146.00–199.00)	.69
Total triglycerides, mg/dl	121.00 (93.00–166.00)	121.00 (93.00–170.00)	117.00 (92.00–161.00)	122.00 (94.00–165.00)	.49
HDL, mg/dl	47.00 (39.00–56.00)	45.00 (39.00–54.00)	47.00 (40.00–56.00)	48.00 (39.00–57.00)	**.04**
LDL, mg/dl	94.00 (74.00–119.00)	97.00 (75.00–120.00)	93.00 (74.00–116.00)	93.00 (74.00–119.00)	.56
Blood glucose monitoring					
Fasting glucose, mg/dl	129.00 (111.00–148.00)	130.00 (111.00–152.00)	129.00 (112.00–146.00)	127.00 (109.00–143.00)	.13
Insulin, μU/mL	12.95 (8.53–19.68)	13.58 (8.60,21.91)	13.14 (8.71,19.25)	12.63 (8.02,18.81)	.24
Insulin resistance index, %	2.56 (4.18–6.88)	2.57 (4.57–7.42)	2.57 (4.35–6.72)	2.51 (3.90–6.40)	.09
HbA1c, %	6.40 (5.90–7.00)	6.60 (6.10–7.30)	6.40 (5.90–6.90)	6.20 (5.80–6.70)	**<.001**
Renal biomarkers					
Creatinine, mg/dL	0.92 (0.77–1.10)	0.95 (0.80–1.19)	0.92 (0.76–1.09)	0.88 (0.75–1.06)	**.001**
Uric acid, mg/dL	5.90 (4.90–6.90)	6.00 (5.10–7.30)	5.90 (4.90–6.80)	5.80 (4.80–6.70)	**.005**
eGFR, mL/min/1.73 m^2^	71.49 (58.86–82.87)	74.89 (60.51–85.84)	70.30 (58.15–82.08)	69.10 (59.38–78.04)	.08
Albuminuria, mg/g Cr	12.07 (6.67–30.19)	15.68 (7.81–41.44)	10.87 (6.42–29.79)	10.65 (6.12–21.28)	**<.001**
Urine creatinine, mg/dL	82.00 (47.00–124.00)	83.50 (48.00–124.00)	81.50 (49.00–124.00)	79.50 (44.00–125.00)	.79
CysC, mg/L	1.13 (0.97–1.35)	1.17 (1.01,1.43)	1.12 (0.97–1.34)	1.09 (0.96–1.28)	**<.001**
Complete blood counts					
WBC, mm^3^	5.90 (5.00–7.00)	6.00 (5.00–7.00)	5.90 (5.00–7.20)	5.80 (5.00–6.80)	.36
RBC, mm^3^	4.44 (4.11–4.78)	4.42 (4.11–4.72)	4.49 (4.13–4.82)	4.44 (4.09,4.76)	.20
PLT, mm^3^	241.00 (201.00–288.00)	244.00 (208.00–291.00)	237.00 (201.00–285.00)	243.00 (197.00–289.00)	**.03**
HCT, %	40.00 (36.90–42.90)	39.90 (36.80–42.40)	40.20 (37.00–43.40)	39.90 (36.8–42.70)	.09
HGB, g/dl	13.20 (12.20–14.10)	13.10 (12.10–13.80)	13.30 (12.30–14.30)	13.20 (12.30–14.10)	.57
MCV, μm^3^	91.00 (87.00–94.00)	90.00 (87.00–94.00)	91.00 (88.00–93.00)	91.00 (87.00–94.00)	.29
MCH, pg	29.90 (28.50–31.20)	29.80 (28.20–31.10)	30.00 (28.40–31.10)	30.00 (28.70–31.40)	.11
MCHC, g/dl	33.10 (32.30–33.70)	33.10 (32.20–33.60)	33.10 (32,40–33.70)	33.10 (32.40–33.70)	.42
RDW, %	14.30 (13.60–15.00)	14.40 (13.80–15.20)	14.20 (13.60–14.90)	14.20 (13.50–14.80)	**.002**
MPV, μm^3^	8.40 (7.90–9.00)	8.40 (7.80–8.90)	8.50 (8.00–9.10)	8.40 (7.90–9.00)	.01
LYM%, %	28.50 (23.50–34.80)	28.60 (22.70–35.50)	28.10 (23.30–33.20)	29.00 (23.80–35.00)	.30
MON%, %	11.20 (8.50–14.60)	11.60 (8.60–14.50)	11.10 (8.40–14.50)	11.00 (8.50–15.00)	.71
GRA%, %	59.30 (51.80–66.20)	59.20 (51.40–65.80)	59.90 (53.20–66.80)	58.90 (51.00–65.60)	.18
Myocardial Injury					
NT‐proBNP, pg/mL	104.80 (55.32–209.55)	105.25 (58.63–228.00)	104.80 (55.17–202.50)	101.40 (53.20–219.10)	.61
Hs‐TnT, ng/mL	0.01 (0.01–0.02)	0.01 (0.01–0.02)	0.01 (0.01–0.02)	0.01 (0.01–0.02)	**<.001**
Inflammatory indicators					
CRP, mg/L	2.27 (1.11–4.42)	2.49 (1.35–5.10)	2.12 (1.08–4.44)	2.12 (0.95–4.17)	**.03**
Thyroid index					
FT4, ng/dl	1.19 (1.07–1.31)	1.195 (1.06–1.32)	1.18 (1.07–1.30)	1.19 (1.08–1.32)	.46
T3, ng/dl	112.80 (100.40–125.70)	112.20 (98.40, 125.70)	112.70 (101.20, 126.00)	113.30 (100.30, 125.20)	.63
TSH, mIU/l	2.17 (1.47–3.38)	2.17 (1.44, 3.35)	2.19 (1.56, 3.46)	2.12 (1.36, 3.33)	.56
Anti‐TPO, IU/ml	11.75 (8.83–18.14)	11.50 (8.83, 17.90)	11.80 (9.02, 17.86)	11.94 (8.59, 18.51)	.73

*Note*: Bold values indicated a *p* value less than .05 with statistic significance.

Abbreviations: anti‐TPO, thyroid peroxidase antibody; afSG, aortic‐femoral arterial stiffness gradient; CRP, high sensitivity C‐reactive protein; CysC, cystatin C; eGFR, estimated glomerular filtration rate; FT4, thyroxine (free); GRA%, granulocyte percentage; HbA1c, glycated hemoglobin; HCT, hematocrit; HDL, high‐density lipoprotein; HGB, hemoglobin; hs‐TnT, high‐sensitivity troponin T; IQR, interquartile range; LDL, low‐density lipoprotein; LYM%, lymphocyte percentage; MCH, mean corpuscular hemoglobin; MCHC, mean corpuscular hemoglobin concentration; MCV, mean cell volume; MON%, monocyte percentage; MPV, mean platelet volume; NT‐proBNP, N‐terminal pro‐B‐type natriuretic peptide; PLT, platelet count; RBC, red blood cell count; RDW, red cell distribution width; T3, triiodothyronine; TSH, thyroid stimulating hormone; WBC, white blood cell count.

^a^ Kruskal–Wallis H test.

**TABLE 2:** Correlations of biomarker levels with cfPWV, faPWV, and afSG on both linear and log–log scales using Spearman rank and Pearson correlations.

Biomarker	cfPWV		Log‐cfPWV		faPWV		Log‐faPWV		afSG		Log‐afSG	
	*R*	*p*	*R* ^2^	*p*	*R*	*p*	*R* ^2^	*p*	*R*	*p*	*R* ^2^	*p*
Lipid metabolism												
Total cholesterol, mg/dl	0.025	.400	0.000	.308	0.052	.089	0.001	.354	−0.008	.806	0.000	.619
Total triglycerides, mg/dl	0.044	.145	0.004	**.041**	0.050	.100	0.001	.216	−0.020	.520	0.001	.261
HDL, mg/dl	−0.068	**.025**	0.004	**.034**	0.035	.253	0.000	.457	0.078	**.013**	0.004	**.030**
LDL, mg/dl	0.032	.297	0.001	.346	0.035	.259	0.000	.735	−0.017	.588	0.000	.462
Blood glucose monitoring												
Fasting glucose, mg/dl	0.081	.007	0.005	**.026**	0.102	**<.001**	0.007	**.006**	−0.040	.195	0.000	.611
Insulin, μU/mL	0.019	.539	0.001	.305	−0.047	.131	0.002	.173	0.001	.149	0.002	.155
Insulin resistance index, %	0.0437	.1578	0.002	.152	−0.013	.668	0.000	.727	−0.056	.068	0.001	.161
HbA1c, %	0.175	**<.001**	0.028	**<.001**	−0.056	.064	0.004	**.044**	−0.193	**<.001**	0.030	**<.001**
Renal biomarkers												
Creatinine, mg/dL	0.062	**.040**	0.003	.087	−0.116	**<.001**	0.013	**<.001**	−0.116	**<.001**	0.010	**.001**
Uric acid, mg/dL	0.016	.591	0.000	.832	−0.014	**<.001**	0.017	**<.001**	−0.088	**.004**	0.005	**.022**
eGFR, mL/min/1.73 m^2^	0.062	.192	0.001	.502	−0.027	.566	0.001	.631	−0.083	.084	0.002	.329
Albuminuria, mg/g Cr	0.202	**<.001**	0.031	**<.001**	0.084	**.008**	0.001	.281	−0.137	**<.001**	0.018	**<.001**
Urine creatinine, mg/dL	−0.016	.592	0.000	.978	−0.063	**.039**	0.005	**.022**	−0.017	.576	0.001	.255
CysC, mg/L	0.066	**.028**	0.003	.070	−0.193	**<.001**	0.023	**<.001**	−0.153	**<.001**	0.016	**<.001**
Complete blood counts												
WBC, mm^3^	0.036	.237	0.002	.109	−0.049	.114	0.000	.543	−0.055	.073	0.003	.079
RBC, mm^3^	0.008	.804	0.002	.154	0.093	**.002**	0.008	**.003**	0.030	.334	0.000	.810
PLT, mm^3^	−0.016	.603	0.000	.398	0.164	**<.001**	0.025	**<.001**	0.081	**.008**	0.003	.064
HCT, %	0.008	.799	0.002	.169	0.149	**<.001**	0.021	**<.001**	0.054	.081	0.001	.234
HGB, g/dl	−0.010	.729	0.000	.591	−0.070	**.021**	0.003	.090	−0.017	.589	0.000	.644
MCV, μm3	−0.010	.756	0.000	.843	0.101	**.001**	0.011	**.001**	0.053	.084	0.003	.066
MCH, pg	−0.029	.342	0.001	.349	0.090	**.003**	0.006	**.011**	0.068	**.027**	0.004	**.035**
MCHC, g/dl	−0.033	.280	0.002	.111	0.030	.326	0.000	.729	0.045	.148	0.002	.111
RDW, %	0.038	.208	0.001	.254	−0.163	**<.001**	0.017	**<.001**	−0.116	**<.001**	0.010	**.002**
MPV, μm^3^	−0.002	.957	0.000	.717	0.013	.684	0.000	.605	−0.005	.864	0.000	.920
LYM%, %	−0.037	.223	0.001	.304	−0.003	.963	0.000	.533	0.030	.331	0.000	.604
MON%, %	0.010	.740	0.000	.712	0.001	.965	0.000	.995	−0.007	.834	0.000	.775
GRA%, %	0.031	.315	0.001	.445	0.005	.885	0.000	.541	−0.025	.424	0.000	.751
Myocardial injury												
NT‐proBNP, pg/mL	0.045	.140	0.003	.069	−0.046	.139	0.003	.102	−0.052	.094	0.005	**.023**
Hs‐TnT, ng/mL	0.128	**<.001**	0.010	**.001**	−0.090	**.003**	0.008	**.003**	−0.155	**<.001**	0.017	**<.001**
Inflammatory indicators												
CRP, mg/L	0.050	.095	0.003	.091	−0.133	**<.001**	0.018	**<.001**	−0.108	**<.001**	0.013	**<.001**
Thyroid index												
FT4, ng/dl	0.000	.995	0.000	.855	0.003	.931	0.000	.513	0.015	.635	0.000	.610
T3, ng/dl	−0.023	.452	0.000	.767	0.034	.263	0.001	.255	0.025	.417	0.000	.767
TSH, mIU/l	0.025	.480	0.000	.720	0.017	.636	0.002	.249	−0.007	.845	0.000	.680
Anti‐TPO, IU/ml	0.004	.842	0.003	.106	−0.013	.689	0.000	.654	0.009	.775	0.001	.264

*Note*: Bold values indicated a *p* value less than .05 with statistic significance.

Abbreviations: afSG, aortic‐femoral arterial stiffness gradient; anti‐TPO, thyroid peroxidase antibody; cfPWV, carotid‐femoral pulse wave velocity; faPWV, femoral‐ankle pulse wave velocity; CRP, high sensitivity C‐reactive protein; CysC, cystatin C; eGFR, estimated glomerular filtration rate; faPWV, femoral‐ankle pulse wave velocity; FT4, thyroxine (free); GRA%, granulocyte percentage; HbA1c, glycated hemoglobin; HCT, hematocrit; HDL, high‐density lipoprotein; HGB, hemoglobin; hs‐TnT, high‐sensitivity troponin T; LDL, low‐density lipoprotein; LYM%, lymphocyte percentage; MCH, mean corpuscular hemoglobin; MCHC, mean corpuscular hemoglobin concentration; MCV, mean cell volume; MON%, monocyte percentage; MPV, mean platelet volume; NT‐proBNP, N‐terminal pro‐B‐type natriuretic peptide; PLT, platelet count; RBC, red blood cell count; RDW, red cell distribution width; T3, triiodothyronine; TSH, thyroid stimulating hormone; WBC, white blood cell count.

### 3.3 Association between cfPWV, faPWV, afSG, and all‐cause mortality

**FIGURE 2:**
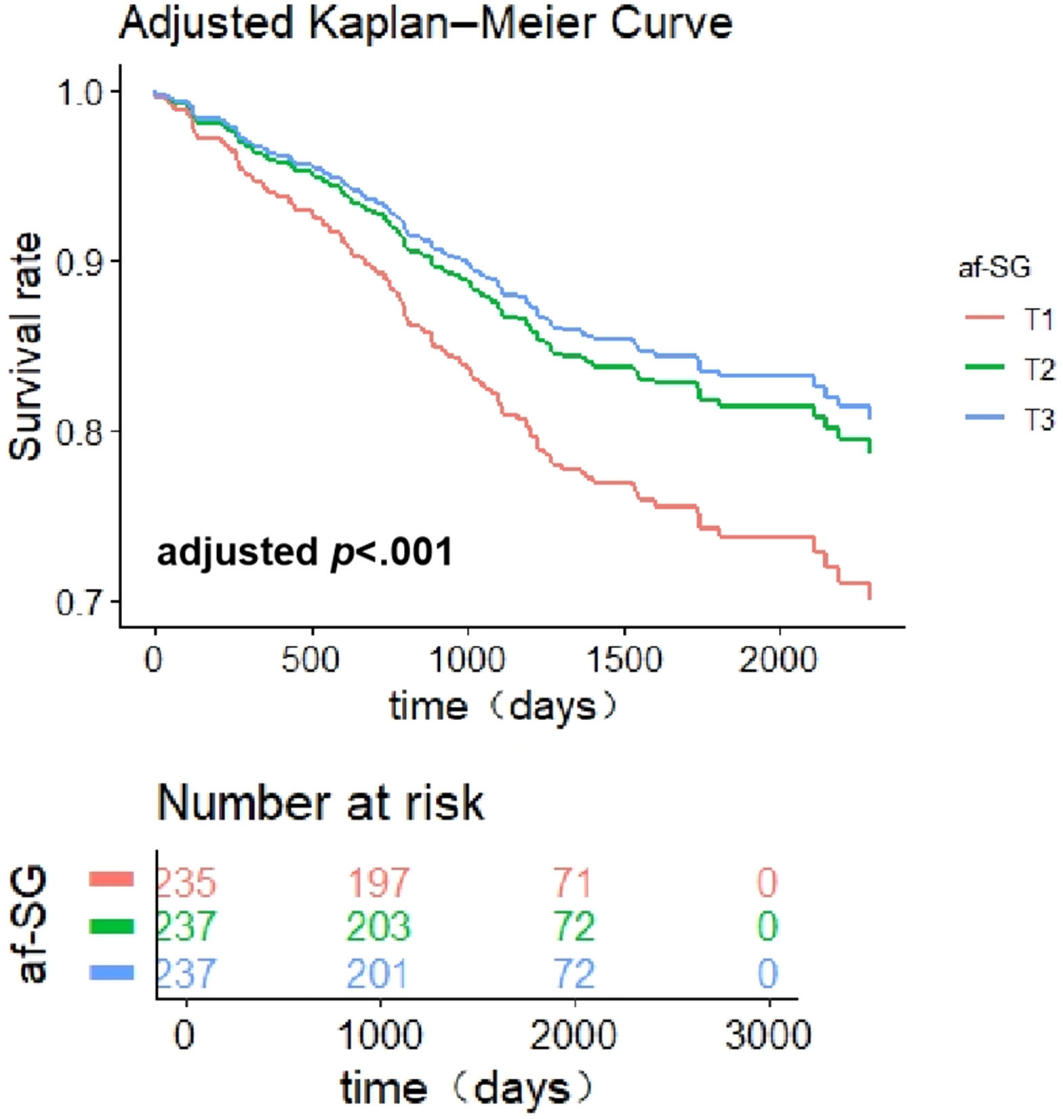
Adjusted Kaplan–Meier survival curves of diabetic patients stratified by the cutoff point of the afSG. afSG, aortic to femoral arterial stiffness gradient; T1, the lowest tertile of afSG; T2, the middle tertile of afSG; T3, the highest tertile of afSG.

We followed 709 diabetic patients for all‐cause mortality, and 131 (18.5%) deaths occurred. Table 3 shows no statistically significant effect for the association of cfPWV and faPWV with all‐cause mortality, but the relationship between all‐cause mortality and afSG was significant after full adjustment (*p* = .018). Compared with the lowest tertile of afSG, the HR (95% CI) of mortality in the highest tertile was 0.543 (0.328–0.900) (*p* for trend = .012). These differences were also presented visibly based on the adjusted Kaplan–Meier survival curves(Figure 2).

**TABLE 3:** Hazard ratios (95% CI) of the association between cfPWV, faPWV, and afSG and all‐cause mortality.

Arterial stiffness	Deaths/*N*	Incidence rate (%)	Crude model		Model 1		Model 2	
			HR (95% CI)	*p* value	HR (95% CI)	*p* value	HR (95% CI)	*p* value
cfPWV (continuous)	131/709	0.011	1.000 (0.999, 1.001)	.220	1.000 (0.999, 1.001)	.473	1.000 (1.000, 1.001)	.028
faPWV (continuous)	131/709	0.011	0.999 (0.998, 1.000)	.229	0.999 (0.998, 1.000)	.186	0.999 (0.998, 1.000)	.082
afSG (continuous)	131/709	0.011	0.728 (0.411, 1.291)	.278	0.786 (0.449, 1.376)	.399	0.548 (0.267, 1.122)	.100
afSG (categorical)								
T1	52/235	0.014	1 (Reference)					
T2	43/237	0.011	0.812 (0.542, 1.217)	.313	0.841 (0.559, 1.265)	.406	0.647 (0.408, 1.024)	.063
T3	38/237	0.009	0.677 (0.442, 1.035)	.072	0.719 (0.467, 1.108)	.135	**0.543 (0.328, 0.900)**	**.018**
*p* for trend				.069		.132		**.012**

*Note*: Model 1: adjusting for age and sex. Model 2: adjusting for age, sex, race, smoking, drinking, body mass index, physical activity, antihypertensive drugs, hypoglycemic drugs, lipid‐lowering drugs, anticoagulants, statins, stroke, myocardial infarction, heart failure, cancer. Bold values indicated a *p* value less than .05 with statistic significance.

Abbreviations: 95% CI, 95% confidence interval; afSG, aortic to femoral arterial stiffness gradient; cfPWV, carotid‐femoral pulse wave velocity; faPWV, femoral‐ankle pulse wave velocity; HR, hazard ratio; T1, the lowest tertile of afSG; T2, the middle tertile of afSG; T3, the highest tertile of afSG.

### 3.4 Sensitivity and subgroup analyses

We conducted a sensitivity analysis to assess whether patients with CVDs or cancer had a confounding effect on the association of afSG and the risk of all‐cause mortality. As shown in Table S2 in Data S1, the correlation of afSG (categories) with all‐cause mortality was not materially changed after excluding those who had a history of CVDs or cancer (*p* for trend = .013, *p* for trend = .012). To confirm whether the correlation between afSG (categories) and all‐cause mortality is robust in different subgroups, subgroup analyses were also performed (Table S3 in Data S1). The association between afSG and all‐cause mortality was more pronounced among women (*p* for interaction = .002). No significant interaction was found in the subgroups of age, race, current smoking, current drinking, history of CVDs, antihypertensive agents, and hypoglycemic drugs. The special results related to cfPWV and faPWV (categories) can be found in Table S4.

## 4 DISCUSSION

In this population‐based study, certain biomarkers including HDL‐C, HbA1c, hs‐TnT, CysC, albuminuria, and creatinine significantly correlated with PWV in diabetic patients. Further, afSG was associated with decreased risk of all‐cause mortality, suggesting it might be an independent predictor of mortality among diabetic individuals.

To our knowledge, few studies have investigated the association of large‐scale biomarkers and PWV among diabetic patients. Previous studies have shown that an increased HDL‐C level is considered an important protective factor against cardiovascular disease.^32^ Our finding suggested that higher HDL‐C may decrease the risk of atherosclerosis, an outcome that was in agreement with prior studies. The underlying mechanisms for the atheroprotective role of HDL‐C may entail reducing the harmful effects of ROS production and increasing endothelial nitric oxide synthase activity.^33^ Recent evidence in agreement with our study reveals that HbA1c was positively correlated with the extent of coronary atherosclerosis lesions.^34^ Moreover, several studies concluded that HbA1c was related to PWV independent of conventional cardiovascular risk factors such as age and blood pressure, a finding that was in line with our results.^35^, ^36^ Various mechanisms have been proposed to link HbA1c with the development of atherosclerosis, including through insulin resistance due to endothelial dysfunction and an abnormal insulin vasodilative effect mediated by endothelium‐derived nitric oxide.^37^ All these alterations may contribute to the development and progression of atherosclerosis. Future studies are needed to investigate the mechanisms underlying the additional effects on the change of HbA1c. Hs‐TnT is considered a predictor of myocardial ischemia.^38^, ^39^ Our results showed that hs‐TnT was correlated with increased arterial stiffness, in line with a previous study confirming the association of PWV with minimally elevated hs‐TnT levels in the elderly and indicating a relationship between central artery stiffness and subclinical myocardial damage.^40^ The underlying mechanism may be that atherosclerosis increased left ventricular end‐systolic pressure and workload, which can lead to left ventricle hypertrophy, diastolic dysfunction, and clinical myocardial injury.^41^

Moreover, we established that certain biomarkers related to renal function (CysC, albuminuria, and creatinine) were positively correlated with cfPWV and negatively correlated with afSG in diabetic patients. For renal biomarkers, our results were consistent with previous studies that demonstrated that CysC was associated with increased arterial stiffness^42^ and can be an independent predictor of arterial stiffness in patients with type 2 diabetes mellitus.^43^ Similar to previous findings, albuminuria was strongly related to endothelial dysfunction in the early phase of atherosclerosis; this relationship was enhanced in subjects with diabetes,^44^ which by itself may predict subclinical atherosclerosis.^45^ Existing literature also shows that longitudinal decline in renal function (creatinine and eGFR) is associated with higher levels of PWV.^46^ The exact mechanism of pathogenesis involved in the relationship between increased arterial stiffness and renal function remains unknown and requires further testing.^47^ However, researchers propose that the decline in renal functioning was associated with atherosclerosis because of endothelial dysfunction, thickening of the media, calcification, and fibrosis, whereas, in turn, arterial stiffness enhanced pressure and flow pulsations to the kidney, thereby inducing microvascular damage.^44^, ^46^

Arterial stiffness has been related to all‐cause and cardiovascular mortality.^48^, ^49^, ^50^ The assessment of aortic arterial stiffness, particularly using cfPWV, has become well established in epidemiological and clinical research settings as a means to determine CVD risk.^49^ However, a notable drawback of cfPWV is its dependence on mean arterial pressure.^12^ Incorporating peripheral arterial stiffness into risk prediction through the aortic‐brachial arterial stiffness gradient (abSG) or afSG has been shown to provide unique and prognostic information beyond cfPWV alone.^11^, ^12^, ^51^, ^52^ However, some argue that the prognostic value and clinical utility of abSG primarily stem from elevated levels of cfPWV.^53^ Additionally, it is worth noting that the upper extremities contribute only a small fraction to the overall arterial system and have limited influence on the absolute hemodynamic load. In contrast, the lower extremities constitute a significant portion of the arterial tree and have a substantial impact on wave reflection patterns and myocardial workload. Several observational studies have evaluated the association of afSG and CVDs or hypertension risk,^11^, ^12^ but no studies have evaluated the association of afSG and all‐cause mortality among diabetic patients. Our research was the first to demonstrate that higher afSG was associated with lower all‐cause mortality in diabetic patients, independent of a wide range of covariates. The main pathological mechanisms were possibly caused by the augmenting wave reflection amplitude, increasing central systolic pressure, and myocardial oxygen consumption plus the formation of AGEs, whose combined effect ultimately leads to cardiovascular and cerebrovascular accident or death.^11^ In this work, only afSG exhibited a significant association between PWV and all‐cause mortality, suggesting that afSG may provide a novel connection between arterial stiffness and all‐cause mortality among diabetic patients and show greater potential than other PWV measurements with certain diseases. However, a number of gaps in the literature remain and need to be addressed in order to ascertain whether the afSG is a clinically viable surrogate endpoint, including but not limited to whether the afSG predicts CVD events and mortality. In addition, our subgroup analysis suggested that the association between afSG and all‐cause mortality was more apparent among females. The primary pathophysiologic mechanism seems to be that the changes of gonadal hormone in elderly women arising from losing the protective effects of endogenous estrogen renders them vulnerable to the progression of arterial stiffness.^54^ Further studies are needed to explore the sex‐specific relationships between artery stiffness and mortality among diabetic population.

The strengths of this research include a comparatively large sample among diabetic patients, a prospective study design, and the investigation of the involvement of other potential confounding factors. In addition, we conducted a comprehensive analysis of the relationship between a panel of biomarkers and PWV and further explored the connection between arterial stiffness with mortality, thereby providing additional reliable evidence for the prevention of carotid atherosclerosis in the diabetic population. Our study inevitably has certain limitations. First, this work is an observational cohort study of the association between biomarkers and PWV, as such, causality in the findings could not be determined. Second, only one‐time measurement was used for each parameter, the relationship between changes in biomarkers over time and PWV require further explorations. Third, we have not taken into account the potential effects of dietary habits. Finally, as this cohort enrolled mainly elder diabetic patients, our results might also not be generalized to younger individuals with diabetes.

## 5 CONCLUSION

Certain biomarkers related to lipid metabolism, blood glucose monitoring, myocardial injury, and renal function are significantly correlated with PWV in diabetic patients. Furthermore, afSG may be an independent predictor of mortality in the diabetic population. Early monitoring of these biomarkers could serve for atherosclerosis prevention, thus reducing the risk of cardiovascular events or death among diabetic individuals. Further prospective studies are warranted to investigate the association between these biomarkers and artery stiffness in the setting of diabetic events.

## AUTHOR CONTRIBUTIONS

Yong‐Qi Liang and Rui Zhou made substantial contributions to conception and design equally and thus are considered co‐first authors. Yong‐Qi Liang conducted statistical analyses, and wrote the manuscript. Rui Zhou provided substantial contributions to the design, analysis, and interpretation of data. Hao‐Wen Chen, Bi‐Fei Cao, Wei‐Dong Fan, Kuan Liu, Qi Zhong, and Yi‐Ning Huang made critical revisions to the manuscript for important intellectual content. Xian‐Bo Wu revised the conception and design of the study, provided guidance for the statistical analysis. Meng‐Chen Zou assisted in the conception and design of the study and provided final approval of the manuscript version for publication. Xian‐Bo Wu is the guarantor of this work and, as such, had full access to all the data in the study and is responsible for data integrity and data analysis accuracy.

## FUNDING INFORMATION

This study was supported by the National Natural Science Foundation of China (Grant No. 82173607), the Guangdong Basic and Applied Basic Research Foundation (Grant No. 2021A1515011684), the Open Project of the Guangdong Provincial Key Laboratory of Tropical Disease Research (Grant No. 2020B1212060042), and the Guangzhou Science and Technology Project (Grant No. 202102080597).

## CONFLICT OF INTEREST STATEMENT

There are no conflicts of interest to declare.

## Supporting information


**Data S1.** Supporting information.

## Data Availability

The data of this study can be requested from the Atherosclerosis Risk in Communities Study (https://biolincc.nhlbi.nih.gov/studies/aric/).
