# Supplementary material for: Associations of blood biomarkers with arterial stiffness in patients with diabetes mellitus: A population‐based study
Source: J Diabetes. 2023 Jun 16;15(10):853–65. doi: 10.1111/1753-0407.13433 (PMC10590681; doi:10.1111/1753-0407.13433)
Supplement: Supplementary file 1 — Data S1. Supporting information. [file JDB-15-853-s001.docx]

**Table S1. Baseline characteristic of diabetic patients in the ARIC study.**

| **Characteristic** | **Total (n=1079)** |
| --- | --- |
| Age, years | 75.18(5.03) |
| Female, % | 640.00(59.31) |
| Race, % |  |
| White | 757.00(70.16) |
| African American | 322.00(29.84) |
| Body mass index (kg/m^2^) | 30.16(5.32) |
| Smoking status, % |  |
| Never | 486.00(45.04) |
| Former | 533.00(49.40) |
| Current | 60.00(5.56) |
| Drinking status, % |  |
| Never | 264.00(24.47) |
| Former | 364.00(33.74) |
| Current | 451.00(41.80) |
| Physical activity |  |
| Yes | 773(71.64) |
| No | 306(28.36) |
| Heart failure, % | 37.00(4.39) |
| Stroke, % | 128.00(15.18) |
| Myocardial infarction, % | 102.00(12.10) |
| Cancer, % | 36.00(3.34) |
| Lipid-lowering drugs, % | 656.00(60.80) |
| Antihypertensive drugs, % | 938.00(86.93) |
| Hypoglycemic drugs, % | 612.00(56.72) |
| [Anticoagulants](javascript:;), % | 59.00(5.47) |
| Statins, % | 678.00(62.84) |
| PWV measurements |  |
| cfPWV, cm/s | 1245.60(388.12) |
| faPWV, cm/s | 1073.23(184.89) |
| afSG, % | 0.93(0.33) |

Data were presented as mean (SD) for continuous variables or N (%) for categorical variables.

Abbreviations: PWV, pulse wave velocity; cfPWV, carotid-femoral pulse wave velocity; faPWV, femoral-ankle pulse wave velocity; afSG, aortic to femoral arterial stiffness gradient.

**Table S2. Association between afSG and all-cause mortality in sensitivity analyses.**

|  | Excluding participants with cardiovascular disease (n =73) | |  | Excluding participants with cancer (n =22) | |
| --- | --- | --- | --- | --- | --- |
| afSG | Model 1  HR (95% CI) | *p* value |  | Model 1  HR (95% CI) | *p* value |
| T1 | 1.000 (*ref.*) |  |  | 1.000 (*ref.*) |  |
| T2 | 0.541(0.324,0.905) | 0.019 |  | 0.647(0.408,1.024) | 0.063 |
| T3 | 0.555(0.328,0.937) | 0.027 |  | 0.543(0.328,0.900) | 0.024 |
| *p* for trend |  | 0.013 |  |  | 0.012 |

Model 1 was fully adjusted for the same covariates as Model 2 in the Table 3.

Hazard ratios (HRs) and 95% confidence intervals (95%CIs) were calculated by Cox proportional hazards models.

Abbreviation: afSG, aortic to femoral arterial stiffness gradient; T1, the lowest tertile of afSG; T2, the middle tertile of afSG; T3, the highest tertile of afSG; HR, hazard ratio; 95%CI, 95% confidence interval.

**Table S3. Association between afSG tertiles and all-cause mortality in subgroup analyses.**

| variables | T1 |  | T2 |  | T3 |  | *p* for interaction |
| --- | --- | --- | --- | --- | --- | --- | --- |
|  | HR (95% CI) |  | HR (95% CI) |  | HR (95% CI) |  |  |
| Age | 1.000(*ref.*) |  |  |  |  |  | 0.142 |
| ≤75 years |  |  | 0.430(0.226,0.819) |  | 0.422(0.218,0.816) |  |  |
| > 75 years |  |  | 1.053(0.524,2.117) |  | 0.794(0.355,1.776) |  |  |
| Sex | 1.000(*ref.*) |  |  |  |  |  | 0.002 |
| Male |  |  | 0.703(0.337,1.470) |  | 1.026(0.492,2.140) |  |  |
| Female |  |  | 0.645(0.357,1.165) |  | 0.329(0.156,0.693) |  |  |
| Race | 1.000(*ref.*) |  |  |  |  |  | 0.795 |
| White |  |  | 0.731(0.411,1.302) |  | 0.616(0.337,1.127) |  |  |
| African American |  |  | 0.692(0.305,1.571) |  | 0.752(0.283,2.000) |  |  |
| Smoking status | 1.000(*ref.*) |  |  |  |  |  | 0.181 |
| Smokers |  |  | 0.868(0.481,1.565) |  | 0.769(0.411,1.439) |  |  |
| Never smokers |  |  | 0.559(0.256,1.219) |  | 0.361(0.147,0.887) |  |  |
| Drinking status | 1.000(*ref.*) |  |  |  |  |  | 0.446 |
| Drinkers |  |  | 0.745(0.431,1.280) |  | 0.726(0.408,1.293) |  |  |
| Never drinkers |  |  | 0.736(0.294,1.845) |  | 0.447(0.151,1.323) |  |  |
| History of CVDs | 1.000(*ref.*) |  |  |  |  |  | 0.146 |
| Yes |  |  | 1.626(0.286,9.256) |  | 0.951(0.104,8.734) |  |  |
| No |  |  | 0.562(0.337,0.935) |  | 0.583(0.347,0.979) |  |  |
| History of hypoglycemic drugs | 1.000(*ref.*) |  |  |  |  |  | 0.222 |
| Yes |  |  | 0.481 (0.249,0.927) |  | （0.249,0.927） |  |  |
| No |  |  | 0.925(0.447,1.915) |  | （0.208,0.853） |  |  |
| History of antihypertensive drugs | 1.000(*ref.*) |  |  |  |  |  | 0.785 |
| Yes |  |  | 0.693(0.423,1.133) |  | 0.693(0.423,1.133) |  |  |
| No |  |  | 0.402(0.069,2.326) |  | 0.608(0.351,1.054) |  |  |

Abbreviation: afSG, aortic to femoral arterial stiffness gradient; CVDs, cardiovascular diseases; T1, the lowest tertile of afSG; T2, the middle tertile of afSG; T3, the highest tertile of afSG; HR, hazard ratio; 95%CI, 95% confidence interval.

**Table S4. Hazard ratios (95% CI) of the association between cfPWV(categorical), faPWV(categorical), and all-cause mortality.**

|  | Deaths/N | Incidence rate（%） |  | Crude model | |  | Model 1 | |  | Model 2 | |
| --- | --- | --- | --- | --- | --- | --- | --- | --- | --- | --- | --- |
|  |  |  |  | HR (95% CI) | *p* value |  | HR (95% CI) | *p* value |  | HR (95% CI) | *p* value |
| cfPWV (categorical) |  |  |  |  |  |  |  |  |  |  |  |
| T1 | 41/237 | 0.010 |  | 1 (Reference) |  |  |  |  |  |  |  |
| T2 | 41/237 | 0.010 |  | 1.013(0.653,1.571) | 0.954 |  | 0.983(0.633,1.526) | 0.938 |  | 1.107(0.663,1.851) | 0.697 |
| T3 | 35/235 | 0.013 |  | 1.304(0.858,1.982) | 0.214 |  | 1,210(0.785,1.863) | 0.388 |  | 1.558(0.940,2.580) | 0.085 |
| fapwv (categorical) |  |  |  |  |  |  |  |  |  |  |  |
| T1 | 49/236 | 0.013 |  | 1 (Reference) |  |  |  |  |  |  |  |
| T2 | 38/237 | 0.009 |  | 0.759(0.495,1.165) | 0.207 |  | 0.757(0.493,1.163) | 0.204 |  | 0.679(0.418,1.104) | 0.119 |
| T3 | 45/236 | 0.012 |  | 0.954(0.634,1.436) | 0.823 |  | 0.941(0.625,1.417) | 0.769 |  | 0.834(0.513,1.357) | 0.466 |

Model 1: adjusting for age and sex.

Model2: adjusting for age, sex, race, smoking, drinking, BMI, physical activity, Antihypertensive drugs, Hypoglycemic drugs, lipid-lowering drugs, [anticoagulants](javascript:;), statins, stroke, myocardial infarction, heart failure, cancer.

Abbreviation: cfPWV, carotid-femoral pulse wave velocity; faPWV, femoral-ankle pulse wave velocity; afSG, aortic to femoral arterial stiffness gradient; T1, the lowest tertile of afSG; T2, the middle tertile of afSG; T3, the highest tertile of afSG; HR, hazard ratio; 95%CI, 95% confidence interval.


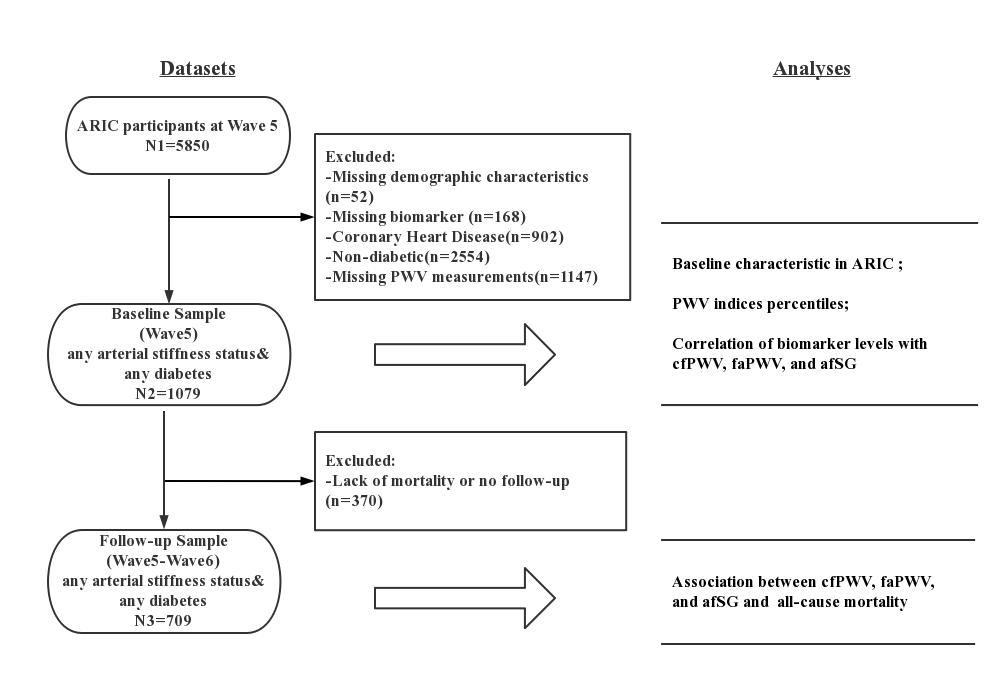


**Figure S1. Flow Chart**

Abbreviation: ARIC, Atherosclerosis Risk in Communities; PWV, pulse wave velocity; cfPWV, carotid-femoral pulse wave velocity; faPWV, femoral-ankle pulse wave velocity; afSG, aortic to femoral arterial stiffness gradient.
